# The genome sequence of the Red-green Carpet, *Chloroclysta siterata* (Hufnagel, 1767)

**DOI:** 10.12688/wellcomeopenres.19301.1

**Published:** 2023-05-10

**Authors:** Douglas Boyes, Peter W.H. Holland, University of Oxford and Wytham Woods Genome Acquisition Lab, Darwin Tree of Life Barcoding collective, Wellcome Sanger Institute Tree of Life programme, Wellcome Sanger Institute Scientific Operations: DNA Pipelines collective, Tree of Life Core Informatics collective, Darwin Tree of Life Consortium

**Affiliations:** 1 UK Centre for Ecology & Hydrology, Wallingford, England, UK; 2 University of Oxford, Oxford, England, UK

**Keywords:** Chloroclysta siterata, Red-green Carpet, genome sequence, chromosomal, Lepidoptera

## Abstract

We present a genome assembly from an individual male *Chloroclysta siterata* (the Red-green Carpet; Arthropoda; Insecta; Lepidoptera; Geometridae). The genome sequence is 437.9 megabases in span. Most of the assembly is scaffolded into 21 chromosomal pseudomolecules including the Z sex chromosome. The mitochondrial genome has also been assembled and is 16.7 kilobases in length. Gene annotation of this assembly on Ensembl identified 11,814 protein coding genes.

## Species taxonomy

Eukaryota; Metazoa; Ecdysozoa; Arthropoda; Hexapoda; Insecta; Pterygota; Neoptera; Endopterygota; Lepidoptera; Glossata; Ditrysia; Geometroidea; Geometridae; Larentiinae; *Chloroclysta*; *Chloroclysta siterata* (Hufnagel, 1767) (NCBI:txid934828).

## Background

The Red-green Carpet, *Chloroclysta siterata*, is a delicately patterned moth in the family Geometridae. The name ‘carpet moth’ for this and closely related species has no relation to their diet; instead, it derives from the forewing markings which comprise a series of irregular bands, considered to resemble the ornate patterns on woven rugs and carpets. In *C. siterata*, these bands are alternating shades of dark and light green, suffused with ruby red streaks. The wings are held flat against the surface at rest and may provide cryptic camouflage on bark and lichen. The green colouration is vivid in freshly emerged specimens but fades with age; this may be the basis for the specific name *siterata*, meaning ‘pertaining to corn’, a plant which also changes colour ( Emmet, 1991)

The species is widespread across northern and eastern Europe, and in the UK is found predominantly in southern England and Wales ( NBN Atlas Partnership, 2021; GBIF Secretariat, 2022). Both distribution and abundance have shown large long-term increases – over six-fold since 1970 ( Randle *et al.*, 2019). *C. siterata* has an unusual life-cycle, being univoltine but with two flight periods. Most records of the adult moth in Britain and Ireland are from September to November, when they will visit ivy blooms or are attracted to light. These adults mate, and the mated females overwinter as adults, emerging to give a second flight period from April to June ( Newman, 1869). In Italy, Austria and Finland, overwintering in caves has been recorded ( Moog *et al.*, 2021; Soderholm, 2022; Teobladelli, 2008). After egg-laying, the larvae develop through July and August, feeding on foliage of deciduous trees - primarily oak but also apple, cherry, rose, rowan, blackthorn and birch ( South, 1961; Waring *et al.*, 2017).

A genome sequence of *C. siterata* will be useful in analyses of molecular adaptations to polyphagy and as part of wider comparative studies into genome evolution in the Lepidoptera.

## Genome sequence report

**Figure 1.**
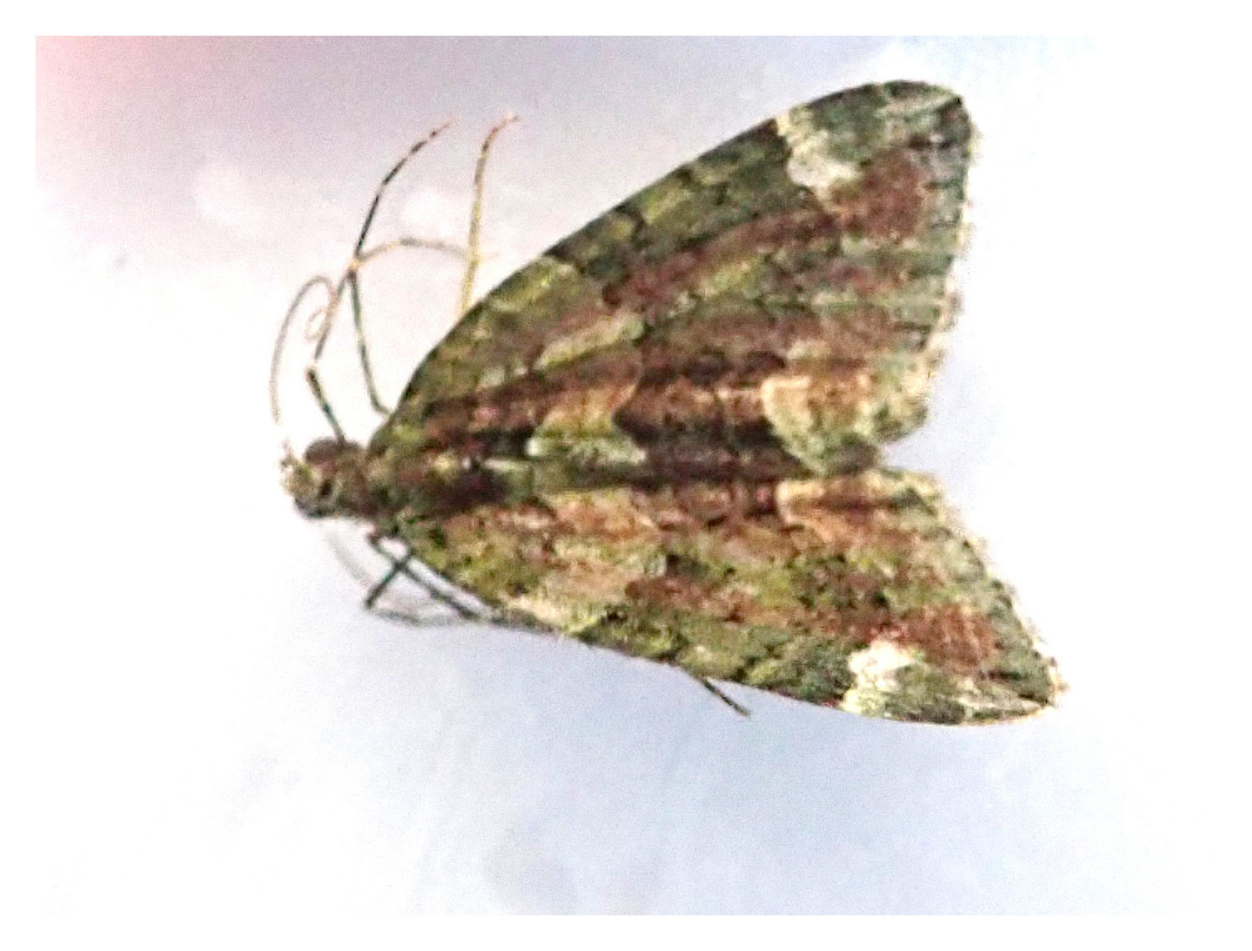
Photographs of the *Chloroclysta siterata* (ilChlSite2) specimen used for genome sequencing.

The genome was sequenced from one male *Chloroclysta siterata* ( Figure 1) collected from Wytham Woods, Oxfordshire (biological vice-county: Berkshire), UK (latitude 51.77, longitude –1.34). A total of 39-fold coverage in Pacific Biosciences single-molecule HiFi long reads was generated. Primary assembly contigs were scaffolded with chromosome conformation Hi-C data. Manual assembly curation corrected two missing joins or mis-joins.

**Figure 2.**
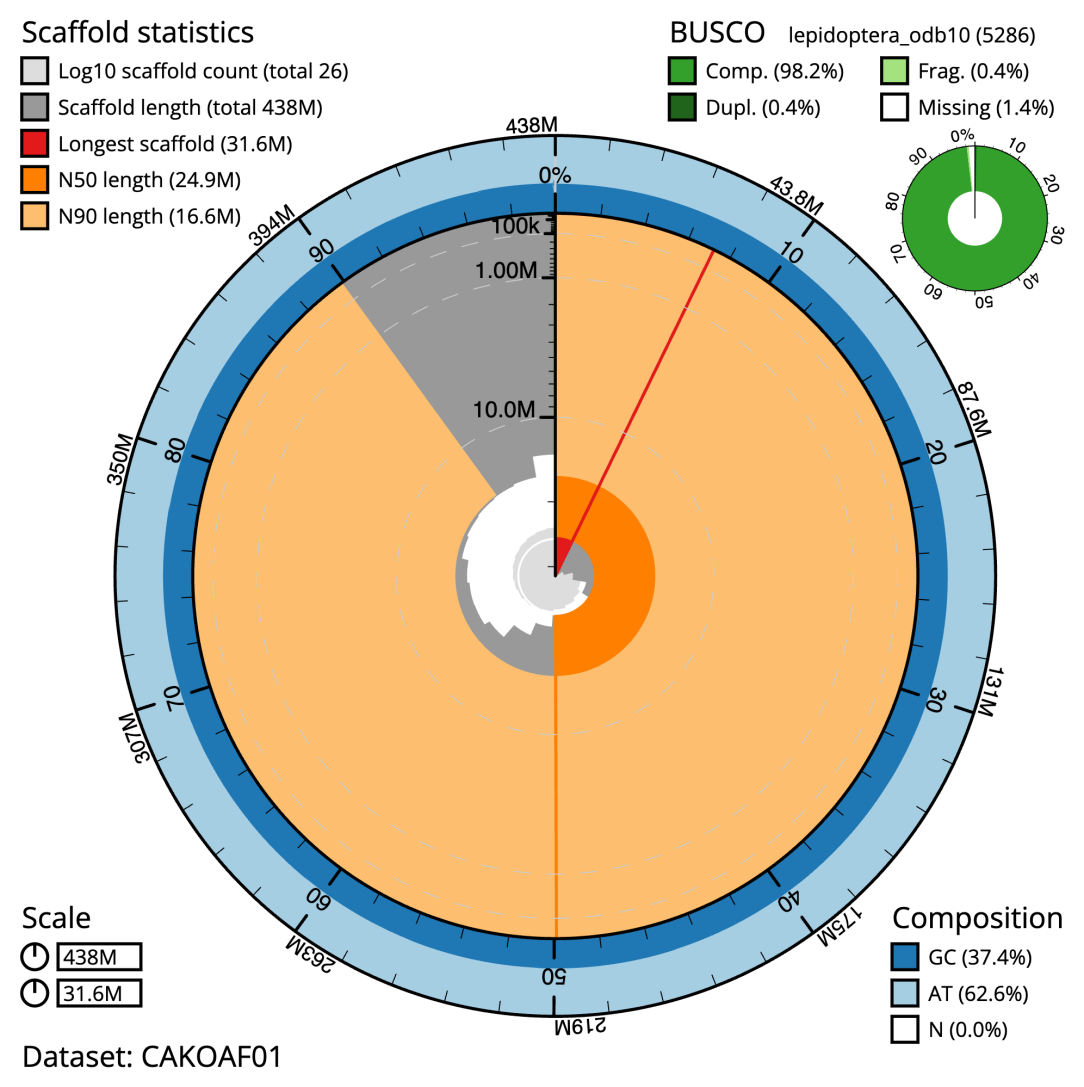
Genome assembly of *Chloroclysta siterata*, ilChlSite2.1: metrics. The BlobToolKit Snailplot shows N50 metrics and BUSCO gene completeness. The main plot is divided into 1,000 size-ordered bins around the circumference with each bin representing 0.1% of the 437,887,824 bp assembly. The distribution of scaffold lengths is shown in dark grey with the plot radius scaled to the longest scaffold present in the assembly (31,593,026 bp, shown in red). Orange and pale-orange arcs show the N50 and N90 scaffold lengths (24,933,739 and 16,571,927 bp), respectively. The pale grey spiral shows the cumulative scaffold count on a log scale with white scale lines showing successive orders of magnitude. The blue and pale-blue area around the outside of the plot shows the distribution of GC, AT and N percentages in the same bins as the inner plot. A summary of complete, fragmented, duplicated and missing BUSCO genes in the lepidoptera_odb10 set is shown in the top right. An interactive version of this figure is available at https://blobtoolkit.genomehubs.org/view/ilChlSite2.1/dataset/CAKOAF01/snail.

The final assembly has a total length of 437.9 Mb in 25 sequence scaffolds with a scaffold N50 of 24.9 Mb ( Table 1). Most (99.97%) of the assembly sequence was assigned to 20 chromosomal-level scaffolds, representing 19 autosomes, and the Z sex chromosome. Chromosome-scale scaffolds confirmed by the Hi-C data are named in order of size ( Figure 2– Figure 5; Table 2). While not fully phased, the assembly deposited is of one haplotype. Contigs corresponding to the second haplotype have also been deposited. The mitochondrial genome was also assembled and can be found as a contig within the multifasta file of the genome submission.

**Table 1.** Genome data for *Chloroclysta siterata*, ilChlSite2.1.

Project accession data		
Assembly identifier	ilChlSite2.1	
Species	*Chloroclysta siterata*	
Specimen	ilChlSite2	
NCBI taxonomy ID	934828	
BioProject	PRJEB50736	
BioSample ID	SAMEA8603199	
Isolate information	ilChlSite2, thorax (genome sequencing), head (Hi-C scaffolding), abdomen (RNA sequencing)	
Assembly metrics *		*Benchmark*
Consensus quality (QV)	70.1	*≥ 50*
*k*-mer completeness	100%	*≥ 95%*
BUSCO **	C:98.2%[S:97.8%,D:0.4%], F:0.4%,M:1.4%,n:5,286	*C ≥ 95%*
Percentage of assembly mapped to chromosomes	99.97%	*≥ 95%*
Sex chromosomes	Z chromosome	*localised homologous pairs*
Organelles	Mitochondrial genome assembled	*complete single alleles*
Raw data accessions		
PacificBiosciences SEQUEL II	ERR8575370, ERR8575371	
Hi-C Illumina	ERR8571652	
PolyA RNA-Seq Illumina	ERR8571653	
Genome assembly		
Assembly accession	GCA_932294275.1	
*Accession of alternate haplotype*	GCA_932294285.1	
Span (Mb)	437.9	
Number of contigs	29	
Contig N50 length (Mb)	20.6	
Number of scaffolds	25	
Scaffold N50 length (Mb)	24.9	
Longest scaffold (Mb)	31.6	
Genome annotation		
Number of protein-coding genes	11,814	
Number of non-coding genes	1,416	
Number of transcripts	19,880	

^*^Assembly metric benchmarks are adapted from column VGP-2020 of “Table 1: Proposed standards and metrics for defining genome assembly quality” from ( Rhie *et al.*, 2021). ^**^BUSCO scores based on the lepidoptera_odb10 BUSCO set using v5.3.2. C = complete [S = single copy, D = duplicated], F = fragmented, M = missing, n = number of orthologues in comparison. A full set of BUSCO scores is available at https://blobtoolkit.genomehubs.org/view/ilChlSite2.1/dataset/CAKOAF01/busco.

**Figure 3.**
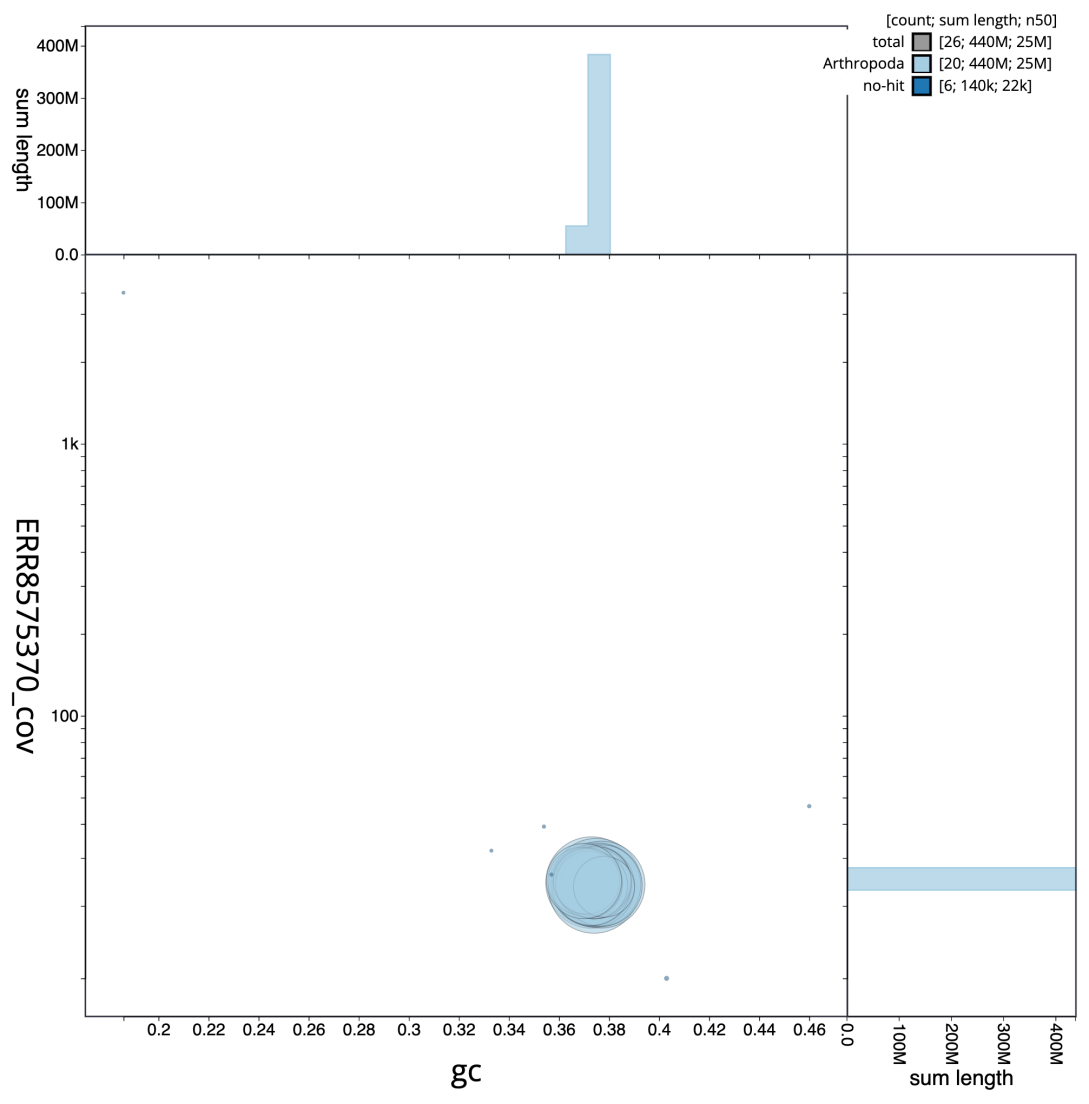
Genome assembly of *Chloroclysta siterata*, ilChlSite2.1: BlobToolKit GC-coverage plot. Scaffolds are coloured by phylum. Circles are sized in proportion to scaffold length. Histograms show the distribution of scaffold length sum along each axis. An interactive version of this figure is available at https://blobtoolkit.genomehubs.org/view/ilChlSite2.1/dataset/CAKOAF01/blob.

**Figure 4.**
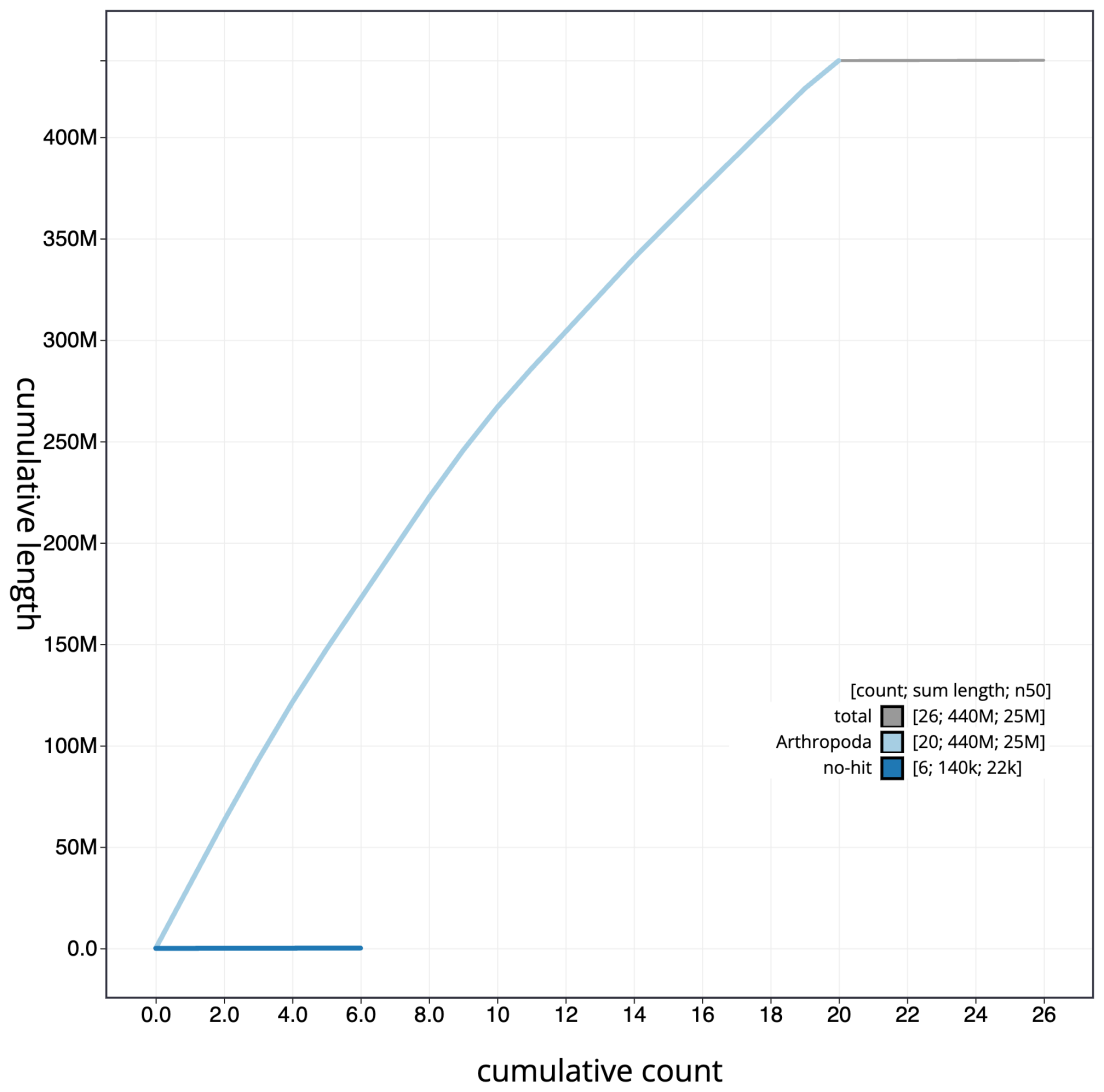
Genome assembly of *Chloroclysta siterata*, ilChlSite2.1: BlobToolKit cumulative sequence plot. The grey line shows cumulative length for all scaffolds. Coloured lines show cumulative lengths of scaffolds assigned to each phylum using the buscogenes taxrule. An interactive version of this figure is available at https://blobtoolkit.genomehubs.org/view/ilChlSite2.1/dataset/CAKOAF01/cumulative.

**Figure 5.**
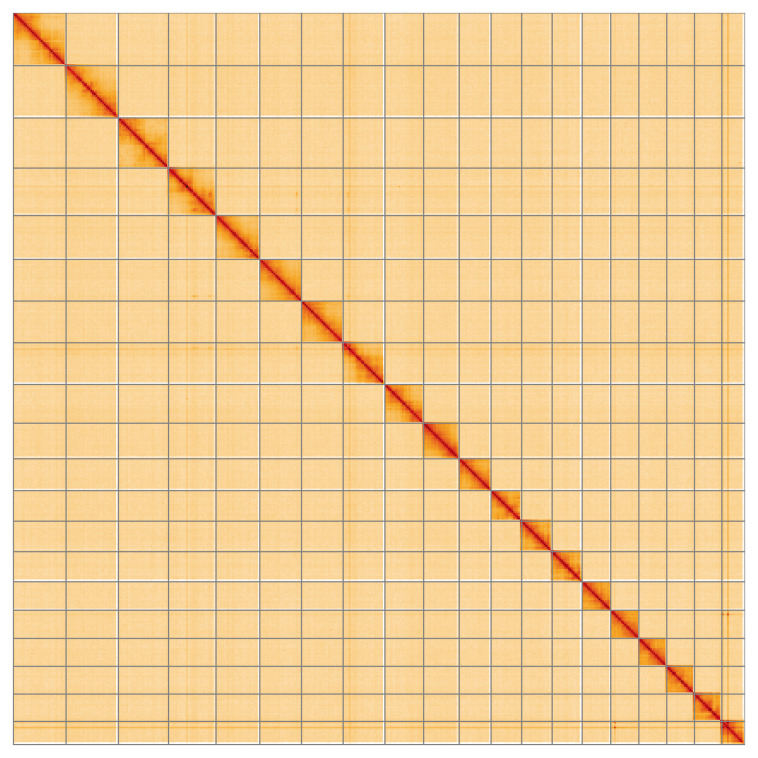
Genome assembly of *Chloroclysta siterata*, ilChlSite2.1: Hi-C contact map of the ilChlSite2.1 assembly, visualised using HiGlass. Chromosomes are shown in order of size from left to right and top to bottom. An interactive version of this figure may be viewed at https://genome-note-higlass.tol.sanger.ac.uk/l/?d=V7ANhS-_Rd6CIbouTb-IJA.

**Table 2.** Chromosomal pseudomolecules in the genome assembly of *Chloroclysta siterata*, ilChlSite2.

INSDC accession	Chromosome	Size (Mb)	GC%
OW028588.1	1	31.59	37.3
OW028589.1	2	31.29	37.4
OW028590.1	3	29.98	37.5
OW028591.1	4	28.42	37.7
OW028592.1	5	26.17	37.6
OW028593.1	6	24.96	37.4
OW028594.1	7	24.94	37.3
OW028595.1	8	24.93	37.7
OW028596.1	9	23.21	37.3
OW028598.1	10	19.06	37.4
OW028599.1	11	18.29	37.5
OW028600.1	12	18.24	37.5
OW028601.1	13	17.95	37.5
OW028602.1	14	17.07	36.9
OW028603.1	15	16.84	37.4
OW028604.1	16	16.7	37.2
OW028605.1	17	16.57	37.2
OW028606.1	18	16.39	37.1
OW028607.1	19	13.87	37.8
OW028597.1	Z	21.26	37
OW028608.1	MT	0.02	18.7

The estimated Quality Value (QV) of the final assembly is 70.1 with *k*-mer completeness of 100%, and the assembly has a BUSCO v5.3.2 ( Manni *et al.*, 2021) completeness of 98.2% (single = 97.8%, duplicated = 0.4%), using the lepidoptera_odb10 reference set ( *n* = 5,286).

Metadata for specimens, spectral estimates, sequencing runs, contaminants and pre-curation assembly statistics can be found at https://links.tol.sanger.ac.uk/species/934828.

## Genome annotation report

The *Chloroclysta siterata* genome assembly GCA_932294275.1 (ilChlSite2.1) was annotated using the Ensembl rapid annotation pipeline ( Table 1; https://rapid.ensembl.org/Chloroclysta_siterata_GCA_932294275.1/Info/Index). The resulting annotation includes 19,880 transcribed mRNAs from 11,814 protein-coding and 1,416 non-coding genes.

## Methods

### Sample acquisition and nucleic acid extraction

A male *Chloroclysta siterata* (ilChlSite2) was collected from Wytham Woods, Oxfordshire (biological vice-county: Berkshire), UK (latitude 51.77, longitude –1.34) on 8 October 2020. The specimen was taken from woodland habitat by Douglas Boyes (University of Oxford) using a light trap. The specimen was identified by the collector and snap-frozen on dry ice.

DNA was extracted at the Tree of Life laboratory, Wellcome Sanger Institute (WSI). The ilChlSite2 sample was weighed and dissected on dry ice with head tissue set aside for Hi-C sequencing. Thorax tissue was cryogenically disrupted to a fine powder using a Covaris cryoPREP Automated Dry Pulveriser, receiving multiple impacts. High molecular weight (HMW) DNA was extracted using the Qiagen MagAttract HMW DNA extraction kit. HMW DNA was sheared into an average fragment size of 12–20 kb in a Megaruptor 3 system with speed setting 30. Sheared DNA was purified by solid-phase reversible immobilisation using AMPure PB beads with a 1.8X ratio of beads to sample to remove the shorter fragments and concentrate the DNA sample. The concentration of the sheared and purified DNA was assessed using a Nanodrop spectrophotometer and Qubit Fluorometer and Qubit dsDNA High Sensitivity Assay kit. Fragment size distribution was evaluated by running the sample on the FemtoPulse system.

RNA was extracted from abdomen tissue of ilChlSite2 in the Tree of Life Laboratory at the WSI using TRIzol, according to the manufacturer’s instructions. RNA was then eluted in 50 μl RNAse-free water and its concentration assessed using a Nanodrop spectrophotometer and Qubit Fluorometer using the Qubit RNA Broad-Range (BR) Assay kit. Analysis of the integrity of the RNA was done using Agilent RNA 6000 Pico Kit and Eukaryotic Total RNA assay.

### Sequencing

Pacific Biosciences HiFi circular consensus DNA sequencing libraries were constructed according to the manufacturers’ instructions. Poly(A) RNA-Seq libraries were constructed using the NEB Ultra II RNA Library Prep kit. DNA and RNA sequencing were performed by the Scientific Operations core at the WSI on Pacific Biosciences SEQUEL II (HiFi) and Illumina HiSeq 4000 (RNA-Seq) instruments. Hi-C data were also generated from head tissue of ilChlSite2 using the Arima v2 kit and sequenced on the Illumina NovaSeq 6000 instrument.

### Genome assembly, curation and evaluation

Assembly was carried out with Hifiasm ( Cheng *et al.*, 2021) and haplotypic duplication was identified and removed with purge_dups ( Guan *et al.*, 2020). The assembly was then scaffolded with Hi-C data ( Rao *et al.*, 2014) using YaHS ( Zhou *et al.*, 2023). The assembly was checked for contamination as described previously ( Howe *et al.*, 2021). Manual curation was performed using HiGlass ( Kerpedjiev *et al.*, 2018) and Pretext ( Harry, 2022). The mitochondrial genome was assembled using MitoHiFi ( Uliano-Silva *et al.*, 2022), which runs MitoFinder ( Allio *et al.*, 2020) or MITOS ( Bernt *et al.*, 2013) and uses these annotations to select the final mitochondrial contig and to ensure the general quality of the sequence. To evaluate the assembly, MerquryFK was used to estimate consensus quality (QV) scores and *k*-mer completeness ( Rhie *et al.*, 2020). The genome was analysed within the BlobToolKit environment ( Challis *et al.*, 2020) and BUSCO scores ( Manni *et al.*, 2021; Simão *et al.*, 2015) were calculated. Table 3 contains a list of software tool versions and sources.

**Table 3.** Software tools: sources and versions.

Software tool	Version	Source
BlobToolKit	4.0.7	https://github.com/blobtoolkit/blobtoolkit
BUSCO	5.3.2	https://gitlab.com/ezlab/busco
Hifiasm	0.16.1-r375	https://github.com/chhylp123/hifiasm
HiGlass	1.11.6	https://github.com/higlass/higlass
Merqury	MerquryFK	https://github.com/thegenemyers/MERQURY.FK
MitoHiFi	2	https://github.com/marcelauliano/MitoHiFi
PretextView	0.2	https://github.com/wtsi-hpag/PretextView
purge_dups	1.2.3	https://github.com/dfguan/purge_dups
YaHS	yahs-1.1.91eebc2	https://github.com/c-zhou/yahs

### Genome annotation

The Ensembl gene annotation system ( Aken *et al.*, 2016) was used to generate annotation for the *Chloroclysta siterata* assembly (GCA_932294275.1). Annotation was created primarily through alignment of transcriptomic data to the genome, with gap filling via protein-to-genome alignments of a select set of proteins from UniProt ( UniProt Consortium, 2019).

### Ethics and compliance issues

The materials that have contributed to this genome note have been supplied by a Darwin Tree of Life Partner. The submission of materials by a Darwin Tree of Life Partner is subject to the Darwin Tree of Life Project Sampling Code of Practice. By agreeing with and signing up to the Sampling Code of Practice, the Darwin Tree of Life Partner agrees they will meet the legal and ethical requirements and standards set out within this document in respect of all samples acquired for, and supplied to, the Darwin Tree of Life Project. All efforts are undertaken to minimise the suffering of animals used for sequencing. Each transfer of samples is further undertaken according to a Research Collaboration Agreement or Material Transfer Agreement entered into by the Darwin Tree of Life Partner, Genome Research Limited (operating as the Wellcome Sanger Institute), and in some circumstances other Darwin Tree of Life collaborators.

## Data Availability

European Nucleotide Archive: *Chloroclysta siterata*. Accession number PRJEB50736; https://identifiers.org/ena.embl/PRJEB50736. ( Wellcome Sanger Institute, 2022) The genome sequence is released openly for reuse. The *Chloroclysta siterata* genome sequencing initiative is part of the Darwin Tree of Life (DToL) project. All raw sequence data and the assembly have been deposited in INSDC databases. Raw data and assembly accession identifiers are reported in Table 1.

## Author information

Members of the University of Oxford and Wytham Woods Genome Acquisition Lab are listed here: https://doi.org/10.5281/zenodo.4789928.

Members of the Darwin Tree of Life Barcoding collective are listed here: https://doi.org/10.5281/zenodo.4893703.

Members of the Wellcome Sanger Institute Tree of Life programme are listed here: https://doi.org/10.5281/zenodo.4783585.

Members of Wellcome Sanger Institute Scientific Operations: DNA Pipelines collective are listed here: https://doi.org/10.5281/zenodo.4790455.

Members of the Tree of Life Core Informatics collective are listed here: https://doi.org/10.5281/zenodo.5013541.

Members of the Darwin Tree of Life Consortium are listed here: https://doi.org/10.5281/zenodo.4783558.
