# Supplementary material for: The genome sequence of the Red-green Carpet, Chloroclysta siterata (Hufnagel, 1767)
Source: Wellcome Open Res. 2023 May 10;8:206. [Version 1] doi: 10.12688/wellcomeopenres.19301.1 (PMC11109689; doi:10.12688/wellcomeopenres.19301.1)
Supplement: Peer review history

# Reviewer report: Reviewer response for version 1

**DOI:** 10.21956/wellcomeopenres.21384.r81602

**Published:** 2024-05-09

**Authors:** Hana Konvičková

**Affiliations:** 1 Institute of Entomology, Biology Centre CAS, Ceske Budejovice, Czech Republic

The paper is clearly written and I appreciate its briefness with no excess information. The authors aimed to sequence a genome of widespread moth *Chloroclysta siterata. *This species is polyphagous and the authors hope to reveal the molecular mechanisms enabling it. They used modern method od HiFi sequencing. The method and processing the data are accurate.

 I have only two minor comments:

 1) Figure 1 - there is only one photograph, although the title says "photographs"

 2) The origin of the sample is mentioned twice (Genome sequence report and Methods), which I believe is not necessary.

Are sufficient details of methods and materials provided to allow replication by others?

Yes

Is the rationale for creating the dataset(s) clearly described?

Yes

Are the datasets clearly presented in a useable and accessible format?

Yes

Are the protocols appropriate and is the work technically sound?

Yes

Reviewer Expertise:

population genetics of animals, with focus on Lepidoptera

I confirm that I have read this submission and believe that I have an appropriate level of expertise to confirm that it is of an acceptable scientific standard.

# Reviewer report: Reviewer response for version 1

**DOI:** 10.21956/wellcomeopenres.21384.r72557

**Published:** 2024-02-20

**Authors:** XiaoLong Liu

**Affiliations:** 1 Hubei University, Wuhan, China

In the Background part, detailed introduction to the morphological characteristics, lifestyle, and distribution range of *Chloroclysta siterata.* Detailed description of the Methods part and the protocols sound appropriate. Clearly described the basic principles of creating datasets. Genome data for *C. siterata* were showed in the table and raw sequence data and the assembly have been deposited in INSDC databases.

Are sufficient details of methods and materials provided to allow replication by others?

Yes

Is the rationale for creating the dataset(s) clearly described?

Yes

Are the datasets clearly presented in a useable and accessible format?

Yes

Are the protocols appropriate and is the work technically sound?

Yes

Reviewer Expertise:

Insect Physiology Biochemistry and Molecular Biology, Transcriptomics

I confirm that I have read this submission and believe that I have an appropriate level of expertise to confirm that it is of an acceptable scientific standard.
